# Prevention of di (2-ethylhexyl) Phthalate-induced Testicular Disturbance in Mice by Co-administration of L-carnitine 

**Published:** 2011-12-22

**Authors:** Zohre Zare, Moslem Mohammadi, Hossein Eimani, Majid Malekzadeh Shafaroudi

**Affiliations:** 1 Department of Anatomy, Shahid Beheshti University of Medical Sciences, Tehran, Iran; 2 Department of Physiology and Pharmacology, Mazandaran University of Medical Sciences, Sari, Iran; 3 Department of Anatomy, Baqiatallah University of Medical Sciences, Tehran, Iran; 4 Department of Anatomy, Mazandaran University of Medical Sciences, Sari, Iran

**Keywords:** L-carnitine, di (2-ethylhexyl) phthalate, Spermatogenesis, Testis

## Abstract

**Background:**

di (2-ethylhexyl) phthalate (DEHP) is widely used in the plastic industry and can induce reproductive toxicity. On the other hand, L-carnitine (LC) plays a crucial role in sperm metabolism and maturation. This study evaluates the effect of LC on body and testis weight, testis tissue, count, motility, viability, morphology, and chromatin quality of epididymal sperm, testicular spermatid number (TSN) per gram testis and daily sperm production (DSP) in LC-treated mice.

**Materials and Methods:**

In this experimental study, adult male NMRI mice (mean age: 4 weeks) were given doses of DEHP and LC by gavaging for 2 weeks. All samples were assessed according to World Health Organization (WHO) criteria. Sperm morphology was assessed using Papanicolaou staining and sperm chromatin quality by aniline-blue staining. The left testes were fixed in Bouinś solution for histological examination and the end slices were stained with hematoxylin and eosin (H&E). The right testes were homogenized, and then TSN and DSP were calculated with an improved Neubauer haemocytometer and respective frames. Paired t-test, ANOVA, and Kruskal-Wallis tests were utilized for data analysis.

**Results:**

Co-administration of DEHP and LC not only prevented significant gains in testicular weight, but also maintained the sperm’s normal morphology and chromatin quality (p<0.05). In addition, LC recovered histological changes, TSN, DSP, and sperm count.

**Conclusion:**

These results demonstrated that oral administration of LC partially or generally protects spermatogenesis from DEHP-toxicity in mice.

## Introduction

There is increasing public concern that environmental toxicants have the potential to impair human fertility due to adverse developmental and reproductive effects being observed in laboratory animals and wildlife after their exposure. Phthalate esters have attracted considerable attention due to their high production volume and use as plasticizers in the plastics industry (1). di (2-ethylhexyl) phthalate (DEHP), one of the most commonly used plasticizers, has been shown to leach out from the finished plastics into the air, water, and ground and thus enter foods (2). The principal route of human exposure to DEHP is oral. After oral exposure, most DEHP is rapidly metabolized in the gut into mono (2-ethylhexyl) phthalate (MEHP), the most toxic metabolite of DEHP (3).

Fredricsson observed that DEHP affected human sperm motility in a dose dependent manner (4). It has also been reported that treatment of laboratory animals with DEHP induced developmental and reproductive toxicity. The reproductive toxicity of DEHP in rats and mice has been characterized by reduction in fertility, litter size, sperm density, motility, and ovarian and testicular weights (5, 6). Yet the mechanisms of the spermatogenic disturbance caused by DEHP remain unclear. The production of free-radicals might disrupt the seminiferous epithelium in DEHP-treated animals (7). In previous reports, co-administration of vitamin B12 (8), A, C and E during DEHP-treatment have effectively protected the testes from the disturbance of spermatogenesis in rats (9, 10). Like vitamins B12, A, C, and E, L-carnitine (LC) is also essential for normal spermatogenesis. LC and acetyl-Lcarnitine (ALC), highly concentrated in the epididymis, both play a key role in sperm metabolism by providing readily available energy for use by spermatozoa, which positively affects sperm motility, maturation, and the spermatogenic process (11). Carnitines also have a protective role against reactive oxygen species (ROS) by exerting antioxidant properties (12). Based on the fundamental roles, numerous clinical trials have attempted to demonstrate a beneficial therapeutic effect of LC and/or ALC when administrated to infertile males with various forms of sperm dysfunction (13-16).

Several *in vitro* studies have documented that carnitines enhance sperm motility when added *in vitro* (17, 18). Therefore, the results of previous studies raise the possibility that simultaneous administration of LC may have some influence on DEHP-induced spermatogenic lesions. The aim of the present study is to determine the possible therapeutic effects of LC on DEHP-induced disturbance of spermatogenesis in mice.

## Materials and Methods

### Animals and treatment

In this experimental study,male NMRI mice were maintained in 22 ± 2°C with a 12 hours light:dark cycle and with access to food and water ad libitum during the course of the study. All animal experiments were approved by the Animal Care and Use Committee of Baqiyatallah University of Medical Sciences.

The mice (mean age: 4-6 weeks) were divided into four groups with ten mice per group which were treated daily by gavage for 2 weeks. Control mice in group I were administered vehicle (100 μl corn oil). Animals in group II were treated with 2g DEHP (CAS NO: 117-81-7, Osaka, Japan)/100 μl corn oil/ kg. Group III received 1mg LC (Sigma-Aldrich Chemie, Italy) GmbH/100 μl deionized water and group IV were treated with a 1mg LC/μl deionized water combination 2g DEHP/100μl corn oil per kg body weight. Our previous study showed that corn oil is ineffective in spermatogenesis (19).

### Body and testis weights

The initial body weight on the first day and final body weight on the termination day (prior to cervical dislocation) of the experiment and testis weights of both sides were recorded for each animal. Testes were trimmed of fat prior to recording their weights. After weighing, the left testis was fixed in Bouinś fluid for histopathological examination, and the right testis was frozen at -20°C, until thawed for daily sperm production analysis.

### Histopathological examination

Fixed testis was embedded in paraffin, sectioned and stained with hematoxylin-eosin (H&E). The histopathlogical specimens were examined under light microscopy. Twelve cross-sections of stage VII-VIII seminiferous tubules from each animal were analyzed by Motic software for tubular diameter (from basal lamina to the other side basal lamina), epithelial height (basal lamina to neck of elongated spermatids), and luminal diameter. In addition, with a ×100 magnification light microscope, the number of sertoli cells and round spermatids were counted for each animal in the 12 cross-sections of stage VII-VIII seminiferous tubules. Data were expressed as the mean of the sertoli cells and round spermatids. These stages were chosen as representative to include morphologically identifiable stages.

### Daily sperm production

The right testis was thawed at room temperature, decapsulated, and homogenized in 2 ml of 0.9 % NaCl solution for 6 minutes using a tissue homogenizer set at low speed. The homogenate was allowed to settle for 1 minute and then gently mixed. After thorough mixing of each sample, the number of homogenization-resistant spermatid heads was counted by a hemocytometer. The testicular spermatid number per gram testis (TSN) was calculated according to the respective formula. DSP was calculated by dividing the TSN by 4.84 (the duration of step 14-16 spermatids in the mouse seminiferous epithelial cycle) (20).

### Sperm parameters

The right cauda epididymis of each animal was minced, suspended in 1 ml of T6 medium that contained 4 mg/ml bovine serum albumin (BSA) for 1 hour at 37°C and 5% CO2.

For the sperm count in the caudal epididymis, 5 μl of the sperm suspension was observed with an optical microscope using a haemocytometer with a cover glass of 0.1 mm thickness. The total number of sperm was counted and the mean was calculated from two counts.

For assessing the sperm motility, 5 μl of sample was placed on a clean microscope slide and covered with a coverslip. Immediately, with ×400 light microscope magnification, 100 sperms were analyzed per sample. Each spermatozoa was classified as: A .rapid linear progressive motility, B .slow or sluggish linear or non-linear motility, C .nonprogressive motility or D .immotile sperm. Percent motile sperm (A+B+C) and percent progressive sperm (A+B) were also calculated.

For studying the sperm morphology, a drop of sperm suspension was smeared onto a clean glass slide. The smear was then air dried and fixed in a mixture of equal parts ethanol and ether. The slides were then stained with Papanicolaou stain. Dried stained slides were scanned under oil immersion (100 objectives) for morphological abnormalities. A total of 100 sperms per sample were classified according to their morphology; such as normal, coiled mid piece, hair pin (a kink at the annulus, usually 180°), bent tail (a kink at the annulus, usually 90°), coiled tail, double head, amorphous head, triangular head, pin head and cytoplasmic droplet. Sperm abnormality was expressed as percent.

For sperm vitality, a drop of sample was put on a clean glass slide and mixed with one droplet of eosin B (0.5% in normal saline). A total of 100 sperm were assessed per animal. Each spermatozoa was classified as motile living sperm, immotile living sperm, or dead sperm.

For the sperm chromatin quality, a drop of sample was smeared, dried, and fixed. Then, slides were stained with 5% aniline blue. For each animal, 100 sperm were counted with 100x objective and classified as low-staining sperm, mid-staining sperm, or high-staining sperm.

### Statistical analysis

Analyses were conducted with SPSS 15.0 for Windows software. Comparison between initial and final body weights was done by paired sample ttest. The viability, motility, progressiveness, and morphology of epididymal sperm and the chromatin quality in treated groups were analyzed by Kruskal- Wallis followed by Mann-Whitney U test. Body and testis weight, sperm count, TSN, DSP, seminiferous tubular and luminal diameter, seminiferous epithelium height, sertoli, and the round spermatid number between groups were compared statistically using analysis of variance (ANOVA), followed by Tukey’s post-hoc test for multiple comparison. Statistical significance was set at p<0.05.

## Results

The mean body weight at the beginning of the experiment was not significantly different among the groups. The mean final body weight significantly decreased in the experimental groups when compared to group I (p<0.05). The mean final body weight increased compared to the mean initial body weight in the experimental groups but this difference was not significant except for group I (Table 1).

The right and left testes weight of the DEHPtreated group (group II) significantly decreased compared to group I (p<0.05). No remarkable alternations in the testes weights of group IV were observed compared to group I (control group). Table 1 summarizes body and testes weights.

### Histopathological examination of testis

Tubular diameter decreased in all experimental groups compared to the control group. The decrease was significant (p<0.05), except for group III. In addition, tubular diameter in group IV significantly increased compared to group II (p<0.05). The seminiferous epithelial height of groups II and IV decreased compared to the control. The decrease was significant (p<0.05) only in group II.

**Table 1:** Body and testis weights


Variable	Group			
	I	II	III	IV

**Initial body weight (g)**	20.2 ± 1.6	19.8 ± 1.8	21.4 ± 0.5	21.2 ± 0.7
**Final body weight (g)**	27.1 ± 3.8	20.9 ± 2.2^*^	23.2 ± 0.8^*^	22.2 ± 1.8^*^
**Right testis weight (mg)**	76 ± 18	51 ± 10^*^	75 ± 7	60 ± 10
**Left testis weight (mg)**	75 ± 7	51 ± 11^*^	75 ± 10	61 ± 12


Data represent mean ± SD ^*^ p < 0.05 compared with group I Group I: Control group Group II: DEHP group Group III: L-carnitine group Group IV: DEHP and L-carnitine group

**Table 2:** Results of histopathological examination


Variable	Group			
	I	II	III	IV

**Seminiferous tubular diameter (μm)**	159 ± 15.6	122.5 ± 14.4^*^	158.7 ± 3.2	139.3 ± 15.2^* #^
**Seminiferous luminal diameter (μm)**	63.1 ± 8	60.6 ± 10.2	59.1 ± 8.3	54 ± 8.8
**Seminiferous epithelial height (μm)**	48.9 ± 4.6	30.7 ± 5.2^*^	50 ± 3.7^#^	42.7 ± 6.5^#^
**Number of sertoli cell/tubule**	23.3 ± 2.2	15.1 ± 1.3^*^	22.4 ± 1.8	17.1 ± 2.4^*^
**Number of round spermatid/tubule**	110.5 ± 8.9	51.8 ± 4.1^*^	112.7 ± 8.8	73.3 ± 22.8^* #^


Data represent mean ± SD ^*^ < 0.05 compared with group I ^#^ p < 0.05 compared with group II Group I: Control group Group II: DEHP group Group III: L-¬carnitine group Group IV: DEHP and L-carnitine group

**Table 3:** Results of sperm count, TSN, and DSP


Variable	Group			
	I	II	III	IV

**Sperm count (× 10^6^/ml)**	3.5 ± 1.26	1.5 ± 0.19^*^	3.18 ± 0.47	2.6 ± 1^#^
**TSN (× 10^6^/g testis)**	83.6 ± 22.7	49.9 ± 22.8^*^	82.8 ± 15.4	64.6 ± 15.7^#^
**DSP (× 10^6^/g testis/day)**	17.1 ± 4.7	10.3 ± 4.7^*^	17 ± 3.8	13.4 ± 3^#^


Data represent mean ± SD ^*^ p < 0.05 compared with group I ^#^ p < 0.05 compared with group II Group I: Control group Group II: DEHP group Group III: L-carnitine group Group IV: DEHP and L-carnitine group

In group IV, epithelial height significantly increased when compared to group II. No significant change was detected in the luminal diameter among the groups (Table 2).

The number of sertoli cells and round spermatids per each seminiferous tubule significantly decreased in both groups II and IV compared to the control group (p<0.05). These parameters in group IV increased compared to group II, but only the increase in the number of round spermatids was significant (p<0.05) (Table 2).

### Daily sperm production

Table 3 shows that the number of homogenization- resistant spermatids and daily sperm production per gram testis were not significantly changed in groups III and IV compared to control values. Those of group II were significantly altered by DEHP exposure (p<0.05). TSN and DSP of group IV significantly increased compared to group II (p<0.05).

### Sperm parameters

Results of the sperm count, percentage of motility, and progressiveness of all groups are summarized in tables 3 and 4. Sperm number, percentage of motility and progressiveness of group III were not significantly altered, and those of group II were significantly diminished when compared to the control group. In group IV, decrease in sperm count, motility (%), and progressiveness (%) were seen in comparison with the control; these alternations were significant (p<0.05) except for the parameter of sperm count. There was a significant increase in the sperm count of group IV as compared with that in the control group (p<0.05).

Data of sperm vitality and chromatin quality analyses are shown in Table 4. Regarding the sperm vitality, the percentage of motile living sperm of groups II and IV significantly reduced and percentage of dead sperm in groups II and IV were significantly increased compared to the control group (p<0.05). No remarkable alternations were observed in the other cases.

Regarding sperm chromatin quality, low-staining sperm (%) of group II was significantly diminished, and those of group III was significantly increased compared to the control group (p<0.05).

**Table 4:** Results of sperm motility, progressive, vitality, and chromatin quality


Variable		Group			
		I	II	III	IV

**Motility (%)**		80 (66-95)	40 (11-60.5) *	69.5 (50-94)	41.5 (29-74) *
**Progressive (%)**		47.75 (35-62)	17.5 (5-35.5) *	39.5 (15-64)	19(12-46) *

**Vitality (%)**	**Motile living sperm**	33.75 (7.5-63)	9 (4-23) *	34.25 (13-67)	14.5 (10-22) *
	**Immotile living sperm**	11 (4-19)	8.5 (5-13)	15.75 (1-37)	11 (4-28)
	**Dead sperm**	50.5 (33-80.5)	81.5 (65-91) *	43 (32-73)	75 (65-81) *

**Chromatin quality (%)**	**Low-staining sperm**	75.5 (62.5-86)	40.5 (36-43) *	90 (81-96) *	78 (60-87) #
	**Mid-staining sperm **	25.5 (12-37)	51 (36-59) *	7.5 (4-15) *	18.5 (12-37) #
	**High-staining sperm**	2 (0-5)	8 (5-22) *	2.5 (0-4)	3 (0-5) #


Data represent median (minimum-maximum) * p < 0.05 compared with group I # p < 0.05 compared with group II Group I: Control group Group II: DEHP group Group III: L-carnitine group Group IV: DEHP and L-carnitine group

**Fig 1:**
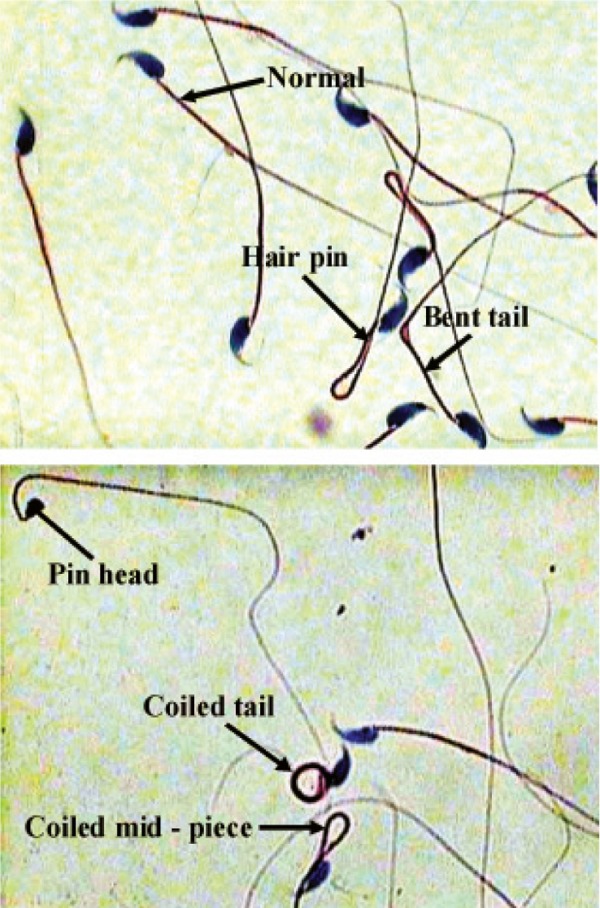
Morphological abnormality of cauda epididymal sperm (Papanicolaou staining, ×1000 magnification).

In group IV, the percentage of low-staining sperm was increased from group I and II, but this increase was significant only in group II (p<0.05). A significant decrease in percentage of mid-staining sperm and high-staining sperm of group IV were seen when compared to group II (p<0.05). No significant changes were detected in these parameters of group IV compared to the control group (Fig 1).

The normal sperm morphology (%) of group II was significantly reduced compared with the control group (p<0.05). There were no remarkable differences among the groups in the other cases.

## Discussion

In this study, we investigated the therapeutic effect of LC on DEHP-induced disruption of spermatogenesis. We found that administration of LC could have some beneficial effects on spermatogenesis in DEHP-treated mice. This shows that LC can protect the testes from the gonadotoxicity of DEHP. This is the first demonstration of the prevention of DEHP-induced spermatogenic injuries by LC.

DEHP was regarded as an endocrine disruptor. Previous reports have demonstrated that DEHP disturbed spermatogenesis (21-24). The mechanism(s) of testicular atrophy induced by DEHP is not known. In previous reports, co-administration of zinc, testosterone or luteinizing hormone-releasing hormone failed to afford protection against DEHP-induced testicular atrophy in young Wistar rats (25-27). Prevention of DEHPinduced disturbance of spermatogenesis was first achieved by administration of vitamin B12 in rats. It is known that vitamin B12 is essential for the synthesis of DNA in all cells that undergo chromosomal replication and division (8). Recently, it has been reported that the administration of vitamins C and E during DEHP-treatment protected rat spermatogenesis from DEHP-gonadotoxicity, and that these vitamins exert a preventive effect through their antioxidant activities (9). Like vitamin B12 and antioxidant vitamins (C and E), LC is also essential for normal spermatogenesis, considering evidence that administration of LC to infertile men with various forms of sperm dysfunction had a beneficial therapeutic effect (13, 15, 16). In addition, LC has a protective role against ROS by exerting antioxidant properties (12).

Recently we reported that oral administration of LC to mice with normal spermatogenesis did not have a significant effect of their reproductive systems (28). A positive correlation has been reported between LC with sperm count and sperm motility (29). Similar findings were reported in a study consisting of 101 infertile men, in which a strong positive relationship between semen LC content with sperm density, sperm motility, and sperm morphology was detected (30).

In our experiment, the analyses showed that administration of LC during DEHP-treatment could generally recover testis weight, normal sperm morphology and sperm chromatin quality and partially protect seminiferous epithelium height, TSN, DSP, sperm count, and number of round spermatid. However body weight, sperm motility, and sperm vitality had not yet reached the control level in spite of LC, and there were still significant differences in comparison with the control group. Therefore, in future experiments, it may be essential that a higher dosage or longer duration of treatment of LC should be determined for complete recovery of DEHP-induced lesions.

## Conclusion

The preventive effects of LC on DEHP-induced testicular damage have been demonstrated for the first time in the present study. Our findings show that LC plays an important role in the maintenance of spermatogenesis.
